# Ezqsar: An R Package for Developing QSAR Models Directly From Structures

**DOI:** 10.2174/1874104501711010212

**Published:** 2017-11-30

**Authors:** Jamal Shamsara

**Affiliations:** Pharmaceutical Research Center, Pharmaceutical Technology Institute, Mashhad University of Medical Sciences, Mashhad, Iran

**Keywords:** Cheminformatics, Lead optimization, MLR, QSAR, R programming language

## Abstract

**Background:**

Quantitative Structure Activity Relationship (QSAR) is a difficult computational chemistry approach for beginner scientists and a time consuming one for even more experienced researchers.

**Method and Materials:**

Ezqsar which is introduced here addresses both the issues. It considers important steps to have a reliable QSAR model. Besides calculation of descriptors using CDK library, highly correlated descriptors are removed, a provided data set is divided to train and test sets, descriptors are selected by a statistical method, statistical parameter for the model are presented and applicability domain is investigated.

**Results:**

Finally, the model can be applied to predict the activities for an extra set of molecules for a purpose of either lead optimization or virtual screening. The performance is demonstrated by an example.

**Conclusion:**

The R package, ezqsar, is freely available *via* https://github.com/shamsaraj/ezqsar, and it runs on Linux and MS-Windows.

## 1 BACKGROUND

Quantitative Structure Activity Relationship (QSAR) is an old but still applicable method for the various branches of chemistry. It attempts to find a model that can predict the biological activity of chemical compounds using their structural features [1]. There are several modules available in commercial tools [2] (SYBYL, MOE and Schrodinger suite) that make QSAR studies simpler than ever.

## 2 MATERIALS AND METHODS

There are also some open source tools available to facilitate the computation of QSAR models. Some of them are collection of individual tools; each performs specific step of a QSAR procedure such as modeling [3] statistical validation [4, 5] and generation of descriptors [6-7]. Some of them present some tools that can be used to generate CoMFA-like 3D QSAR models such as, Open3DQSAR or PyCoMFA [8]. CORAL is a freeware that is developed to build QSAR PLS models using a specific set of descriptors so-called SMILES notation based optimal descriptors [9-10] QSARINS is another standalone freeware that can build QSAR MLR with various functions including data processing and partitioning, validation of the model, prediction of a new compound activity and determination of applicability [11]. However, it does not have any built-in descriptor generation ability. Among the available open source tools, the most similar one to ezqsar is another R-package that is called camb [12]. Some of the features are similar between those packages. They are intended to be used mostly by beginners. They provide a single function that can do the entire job starting with a data set and ends with a complete QSAR model that includes descriptor generation, data processing, modeling, internal and external validity assessment, presentation of a QSAR model with the ability to predict the activities of new structures. The advantages of ezqsar over camb are the applicability domain functionality, easier use and interpretability of the developed model. However, the camb package currently has more training options than ezqsar such as machine learning methods. The advanced users are advised to use caret package to model pre-generated descriptors. It wrapped up QSAR tools in several functions and user can tune several parameters for each one, but ezqsar could be used by advanced users to provide an easy and precise look on the modelability of a data set and prediction of the activity of a test set with estimation of applicability domain. They could also use the power of R scripting to enrich the output plots or automate model building for large number of data set or develop several models for a single data set and discover the best model among them. Finally, the selected descriptors by ezqsar for a reliable QSAR model could be used for mechanistic interpretation of the model.

Here, an open source R (R: a language and environment for statistical computing; R Foundation for Statistical Computing, Vienna, Austria; URL http://www.R-project.org/.) package is introduced that could develop Multiple Linear Regression (MLR) QSAR models from 2D or 3D structures and their corresponding activities *via* a single line command. Then, in an iterative process, the QSAR model could be refined by modifications in, for example, the number of selected descriptors and test set selection. Data set selection and preparation is a first step and the most important step in a QSAR study. The structures should be checked if they are retrieved from public databases. Data set should have the least possible experimental uncertainty. Experimental uncertainty arise from systematic error or in case of single point activity determination. The detection of possible experimental uncertainty in the data set may be detected by statistical methods but it is not easy [13-15]. The descriptor generation in ezqsar is done using CDK library [16]. It computes 2D and 3D descriptors. They are classified into five groups “topological”, “geometrical” “hybrid”, “constitutional”, and “electronic”. If the input structures are in 3D coordinates, the 3D descriptors will be calculated otherwise, the value for the 3D descriptors would be zero. A list of the all-275 CDK descriptors is presented in Table (**1**). Now, ezqsar only accepts SDF file as an input and the structures should be verified beforehand regarding specific chirality, protonation state and tautomeric form.

MLR is a simple, reproducible and easy interpretable method used usually in QSAR. An MLR equation can be expressed generally like the following [17, 18]:

(1) $$Y=a_{0}+a_{1}\times X_{1}+X_{2}+a_{3}\times X_{3}+...+a_{n}\times X_{n}$$

In the above expression, *Y* is the dependent variable (here is activity), *X_1_, X_2_, ..., X_n_* are independent variables (descriptors) present in the model with the corresponding regression coefficients *a_1_, a_2_, ..., a_n_* , respectively, and a_0_ is the constant term of the model. The quality of a MLR model is evaluated using the number of metrics as described below [17, 18].

ezqsar_f function uses Leave-one-out (LOO) cross-validation method for cross-validation:

(2) $$Q^{2}=1-\frac{\sum {\left(Y_{\mathrm{obs}\left(\mathrm{tarin}\right)}-Y_{\mathrm{pred}\left(\mathrm{train}\right)}\right)}^{2}}{\sum {\left(Y_{\mathrm{obs}\left(\mathrm{tarin}\right)}-{\bar{Y}}_{\mathrm{training}}\right)}^{2}}$$

The determination coefficient (*R^2^*) and predictive *R^2^*(*R^2^_pred_*) are defined in the following manners:

(3) $$R^{2}=1-\frac{\sum {\left(Y_{\mathrm{obs}\left(\mathrm{tarin}\right)}-Y_{\mathrm{calc}\left(\mathrm{train}\right)}\right)}^{2}}{\sum {\left(Y_{\mathrm{obs}\left(\mathrm{tarin}\right)}-{\bar{Y}}_{\mathrm{obs}\left(\mathrm{train}\right)}\right)}^{2}}$$

(4) $$R_{\mathrm{pred}}^{2}=1-\frac{\sum {\left(Y_{\mathrm{obs}\left(\mathrm{test}\right)}-Y_{\mathrm{pred}\left(\mathrm{test}\right)}\right)}^{2}}{\sum {\left(Y_{\mathrm{obs}\left(\mathrm{test}\right)}-{\bar{Y}}_{\mathrm{training}}\right)}^{2}}$$

In equations 2-4, *Y_obs(train)_* is the observed activity for the train set, *Y_pred (train)_* is the predicted activity of the training set molecules based on the LOO technique, *Y_calc(train)_* is the model-derived calculated response for the train set and ${\bar{Y}}_{\mathrm{obs}\left(\mathrm{train}\right)}$ is the average of the observed response values for the train set, *Y_obs (test)_* and Y_pred (test)_ are the observed and predicted activity data for the test set compounds, respectively.

The ability of the model to predict activity of the present and other set can be accessed *via* *R^2^* and *Q^2^*, respectively. If the difference between *Q^2^* and *R^2^* is more than 0.3, an overtrained model can be implied. The predictivity of the model also can be assessed by *R^2^_pred_*. Generally, the following criteria roughly indicate a reliable model [17, 19, 20]:

(5) $$\left\{\begin{array}{c} Q^{2}>0.5\\ R^{2}>0.8\\ R_{\mathrm{pred}}^{2}>0.6 \end{array}\right.$$

The applicability domain is defined as a space constructed (structural, chemical, *etc.*) by the model that plays a crucial role for estimating the applicability of the model for predicting the activity of new compounds [17]. In other word, the prediction of a property of interest (here, activity) of a new compound using a QSAR model is applicable only if it is within the applicability domain of the QSAR model. The final model could be used to predict the activity of an extra set of compounds that are within the applicability domain of the model. This is checked by finding possible outliers of the standardized values of the descriptors (*S_ki_*) which are present in the model as well as by calculation of Tanimoto similarity index of a compound fingerprint to the each compound in the train set.

Standardized descriptor values can be calculated as follows, that is a simplified form suggested by Roy K. *et al.* [21]

(6) $$S_{\mathrm{ki}}=\frac{\left|X_{\mathrm{ki}}-{\bar{X}}_{i}\right|}{\sigma_{\mathrm{Xi}}}$$

Where, *K* is the number of compounds, *i* is the number of descriptors, *S_Ki_* is the standardized descriptor *i* for compound *K* (from the training or test set, *X_Ki_* is the original descriptor *i* for compound *K* (from the training or test set), ${\bar{X}}_{i}$ is the mean value of the descriptor *X_i_* for the training set compounds only, *σ_xi_* is the standard deviation of the descriptor *X_i_* for the training set compounds only. The above calculation is meant for all descriptor values present in the model (number of compounds × number of descriptors).

Tanimoto similarity indexes are calculated as it follows [22-24]:

(7) $$\mathrm{Tanimoto}\left(a,b\right)=\frac{N_{\mathrm{ab}}}{\left(N_{a}+N_{b}\right)-N_{\mathrm{ab}}}$$

where *N_ab_* is the number of common “1” bits that occur in both fingerprint a and fingerprint b, *N_a_* is the number of “1” bits in fingerprint a, *N_b_* is the number of “1” bits in fingerprint b. In ezqsar_f, Tanimoto index is computed by fingerprint package.

## 3 IMPLEMENTATION

The codes were implemented in a package available at github and can be installed and loaded by the following commands in R environment:

- install.packages(“devtools “)
- devtools::install_github(“shamsaraj/ezqsar”)
- library (“ezqsar”) #This will load the package

It depends on four packages: caret, fingerprint, leaps and rcdk.

## 4 RESULTS AND DISCUSSION

**Fig. (1):**
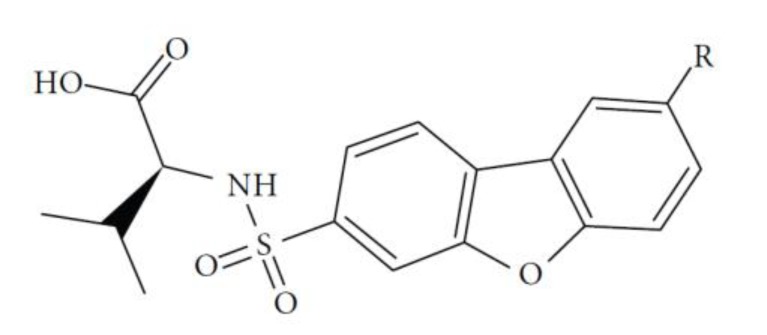
General structure for the dataset.

The performance of the ezqsar package in an example data set that is provided by the package after installation was demonstrated in the study. The data set Table (**1** and Fig. **1**) was taken from a study [25] and a HQSAR model was already available to them [26]. It has a single function called “ezqsar_f”. Like other R functions, one can get help for the function by:

- help(“ezqsar_f”)

An overview of the ezqsar_f workflow is demonstrated in Fig. (**2**). All of the molecules were collected in a single SDF file. Activities were provided in a separate csv file rank ordered same as the SDF file. The activities were expressed as pIC_50,_ however, they also can be expressed as IC_50_. Alternatively, the activities also can be provided in a SDF file with the header of “IC_50_”. Data set contains 24 MMP-12 inhibitors. There is an extra test set that includes three other MMP-12 inhibitors with similar structures. The example can be run by following the command after loading the installed package, ezqsar:

- example (“ezqsar_f”)

This will run following the script:

- file1<-system.file (“extdata”, “molecules-3d.sdf”, package = “ezqsar”)
- file2<-system.file (“extdata”, “IC50.csv”, package = “ezqsar”)
- file3<-system.file (“extdata”, “newset-3d.sdf”, package = “ezqsar”)
- model<-ezqsar_f (SDFfile=file1, activityfile=file2, newdataset=file3, testset=c(4,6,12,22)
- attributes (model)
- print (model$Q2)
- print (model$R2)
- print (model$test)
- print (model$R2_pred)
- print (model$Tanimoto_test_sum)
- print (model$AD_outlier_test)
- print (model$newset)
- print (model$Tanimoto_newset_sum)

The input files were stored as files 1, 2 and 3 by executing lines 1, 2 and 3. The model is developed by executing line 4. This runs the function with default parameters and stores the results as an object called, model. Highly correlated descriptors (correlation coefficient over a defined threshold) can be removed from the descriptor table before further processing. By default, this threshold is 1 (correlated descriptors are not removed), test set ratio is 20% and the selection is based on the activities; descriptors are selected by forward selecting method before final MLR model development. The calculated descriptors are reported as an csv file named “descriptors.csv”. A plot (“Plot-MLR.pdf “) will also be available in the working directory (can be changed by setwd () in R) after a successful run regarding the observed vs predicted activity values for both train and test sets using the developed model Fig. (**3**). Available outputs are shown after executing line 6 and some of the most important ones were printed out by remaining lines of the example (lines 6 to 13). The statistical metrics for the example data set are shown in (Table **2**).

The ezqsar_f function has an ability to perform prediction on up to two extra test sets using the developed model. It could be helpful for either lead optimization process or a ligand based virtual screening.

Different test set selection will lead to different descriptor selection and finally a different model. By running ezqsar function over different test sets, a model with better statistical metrics can be achieved. Here is a sample script:

- file1<-system.file (“extdata”, “molecules-3d.sdf”, package = “ezqsar”)
- file2<-system.file (“extdata”, “IC50.csv”, package = “ezqsar”)
- file3<-system.file (“extdata”, “newset-3d.sdf”, package = “ezqsar”)
- for (h in 1:100){
- set.seed(h)# for each seed number a different test set is selected based on the activities
- model<-ezqsar_f (SDFfile=file1, activityfile=file2, newdataset=file3)
- if ((model$Q2>0.5) & (model$R2>0.8) & (model$R2_pred>0.6)) {
- print (h)
- print (model$Q2)
- print (model$R2)
- print (model$R2_pred
- print(model$Model)
- print (model$Tanimoto_test_sum)
- print (model$AD_outlier_test)
- print (model$newset)
- n1<-(abs(as.numeric(model$newset [1, 2]) - 1.74))^2
- n2<-(abs(as.numeric(model$newset [2, 2]) - 3.39))^2
- n3<-(abs(as.numeric(model$newset [3, 2]) - 2.14))^2
- PRESS<-sum(n1,n2,n3)
- print (PRESS)
- print (model$AD_outlier_newset)
- print(“******************************************”)
- }
- }
- print (c(1.74,3.39,2.14))# observed activities (pIC_50_) for the new set compounds
- print (model$Tanimoto_newset_sum)

By running the script, only the models within the criteria (equation 4) are listed among 100 developed models. To demonstrate the importance of applicability domain, performance of the two model with proper statistical metrics were compared. The results indicated that a QSAR model Table (**2**, model3), despite good statistical metrics (similar to model2), may fail to predict the activity of even structurally related structures if the descriptor values of the extra test set are significantly out of range (in this case, standardized value of one of the descriptors = 8.5).

## CONCLUSION

In conclusion, ezqsar offers a highly useful, easy-to-use tool for QSAR analysis, including calculations of descriptors and applicability domain determination. The package could be extended extending the use of other statistical methods such as Partial Least Squares (PLS) or machine learning methods to develop various QSAR models.

## Figures and Tables

**Fig. (2):**
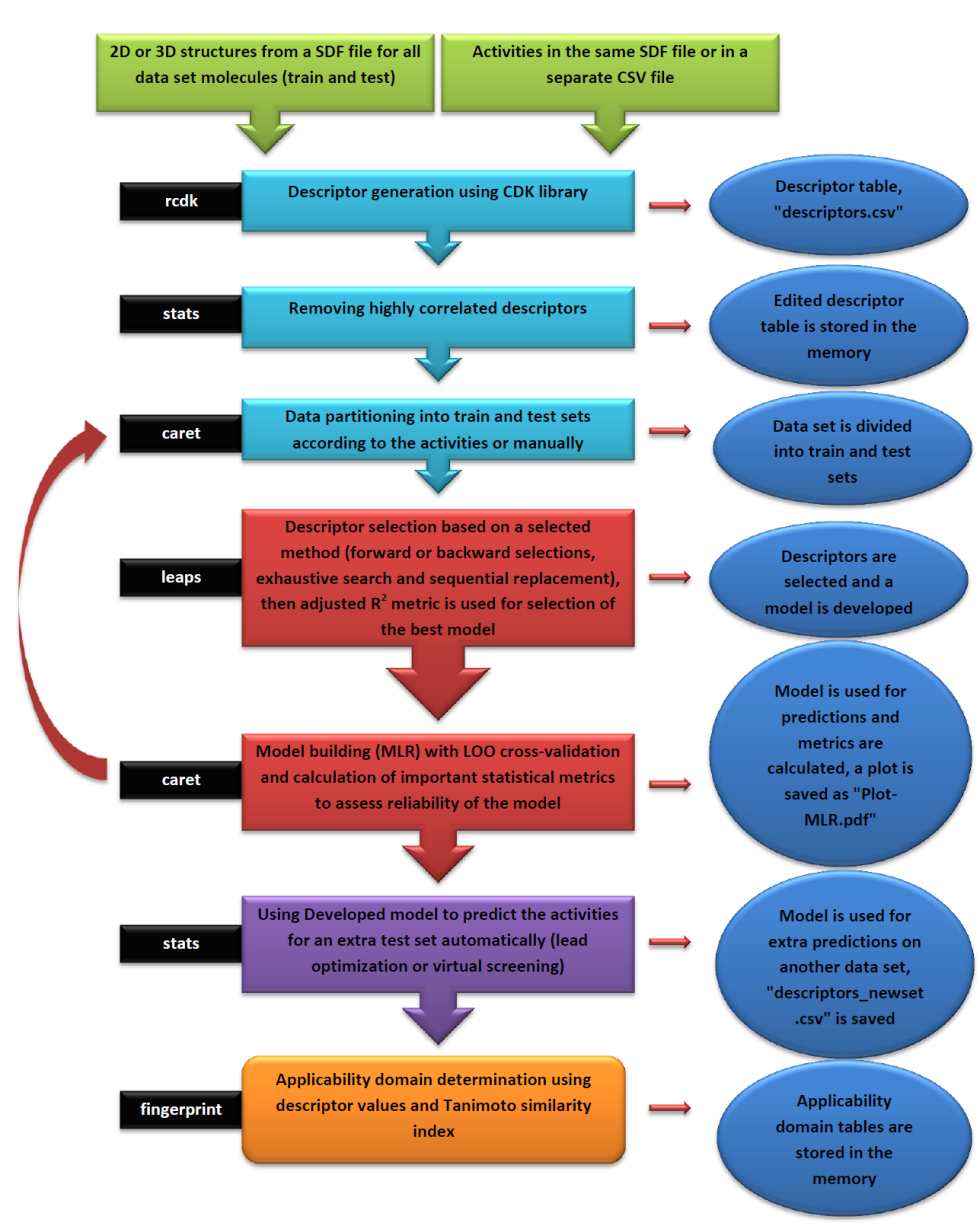
The workflow of ezqsar_f function from ezqsar package.

**Fig. (3):**
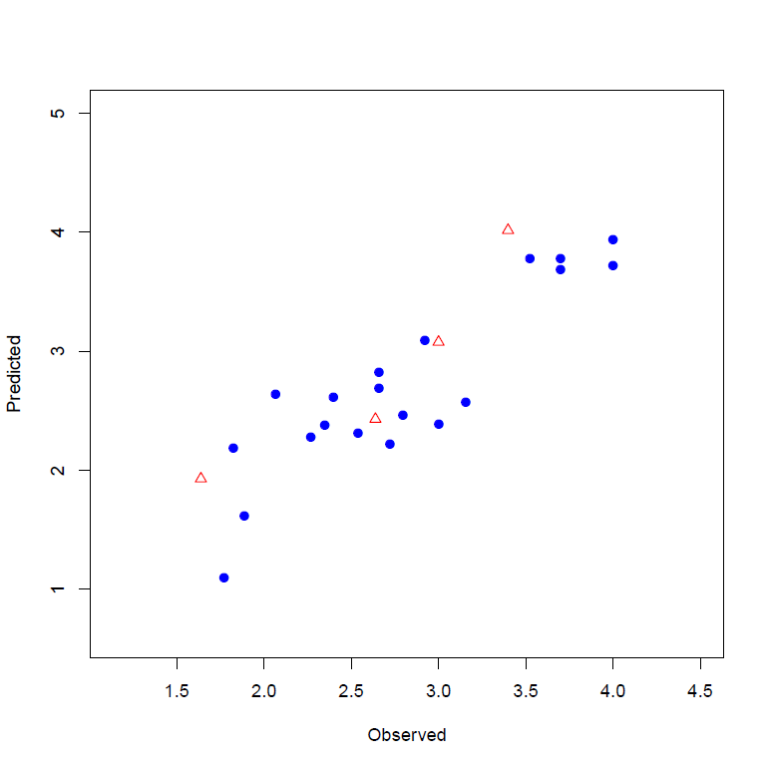
Plot of observed versus predicted activities obtained from model1 for training (blue circles) and test (red triangles) sets.

**Table 1:** Observed and predicted activities of the training, test and new test sets based on the model1. Activities were shown as pIC_50_ (µM). ^a^: Test set, ^b^: new test set. They are provided as an example data set in the package.

Name	R	Observed pIC_50_	Predicted pIC_50_ values	Residues
n1	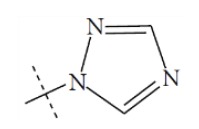	1.89	1.62	0.27
n2	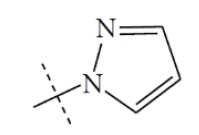	1.82	2.19	-0.37
n3	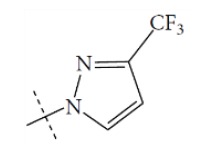	3.15	2.57	0.58
n4^a^	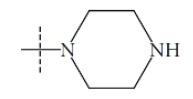	1.64	1.93	-0.29
n5^b^	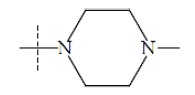	1.74	1.67	0.07
n6	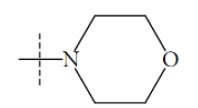	2.66	2.82	-0.16
n7 ^a^	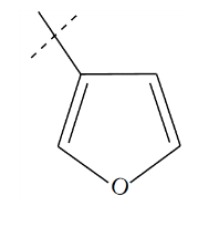	3.40	4.02	-0.62
n8	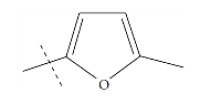	4.00	3.72	0.28
n9	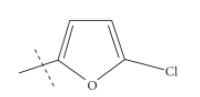	4.00	3.94	0.06
n10	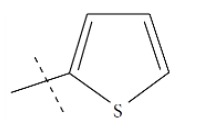	3.70	3.78	-0.08
n11	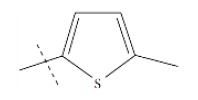	3.70	3.69	0.01
n12	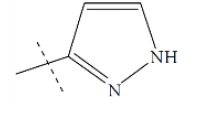	3.00	2.39	0.61
n13 ^b^	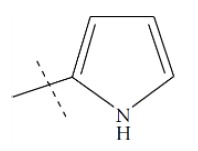	3.39	3.06	0.33
n14 ^a^	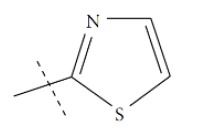	3.00	3.08	-0.08
n15	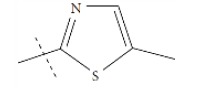	2.92	3.10	-0.17
n16	Methyl	2.07	2.64	-0.58
n17	Ethyl	2.54	2.31	0.22
n18	i-Propyl	2.35	2.38	-0.03
n19	t-Butyl	1.77	1.10	0.67
n20	i-Butyl	2.27	2.28	-0.01
n21	CH2OCH3	2.72	2.22	0.50
n22	CF3	2.66	2.69	-0.04
n23	Cyclopropyl	2.80	2.46	0.33
n24 ^a^	Cyclobutyl	2.64	2.43	0.21
n25 ^b^	Cyclohexyl	2.14	2.48	-0.34
n26	Phenyl	2.40	2.62	-0.22
n27	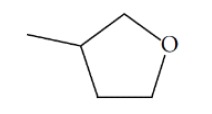	3.52	3.78	-0.26

**Table 2:** Output summary for three models.

Model	Model1	Model2	Model3
Test set compounds	n4,n7,n14,n24	n10, n20, n23, n24	n4, n9, n12, n24
Q2	0.796	0.722	0.858
R2	0.901	0.865	0.941
R2pred	0.708	0.637	0.617
New set compounds and activities	n5=1.74, n13=3.39, n25=2.14	n5=1.74, n13=3.39, n25=2.14	n5=1.74, n13=3.39, n25=2.14
Predicted activities for the new set compounds	n5=1.67, n13=3.06, n25=2.47	n5=1.35, n13=3.00, n25=2.44	n5=1.37, n13=2.78, n25=3.54
predicted residual sum of squares (PRESS)	0.226	0.401	2.480
comment	All of the model descriptors of all compounds have standardized values <3	All of the model descriptors of all compounds have standardized values <3	Standardized value of one of the model descriptors for n25 is very high (= 8.63)

## References

[1] Lewis R.A., Wood D. (2014). Modern 2D QSAR for drug discovery.. Wiley Interdiscip. Rev. Comput. Mol. Sci..

[2] Yee L.C., Wei Y.C. (2012). Current Modeling Methods Used in QSAR/QSPR.. In Statistical Modelling of Molecular Descriptors in QSAR/QSPR, Wiley-VCH Verlag GmbH & Co. KGaA:.

[3] Teófilo R.F., Martins J.P., Ferreira M.M. (2009). Sorting variables by using informative vectors as a strategy for feature selection in multivariate regression.. J. Chemometr..

[4] Baumann D., Baumann K. (2014). Reliable estimation of prediction errors for QSAR models under model uncertainty using double cross-validation.. J. Cheminform..

[5] Roy K., Ambure P. (2016). The “double cross-validation” software tool for MLR QSAR model development.. Chemom. Intell. Lab. Syst..

[6] Dong J., Cao D-S., Miao H-Y., Liu S., Deng B-C., Yun Y-H., Wang N-N., Lu A-P., Zeng W-B., Chen A.F. (2015). ChemDes: an integrated web-based platform for molecular descriptor and fingerprint computation.. J. Cheminform..

[7] Tetko I.V., Gasteiger J., Todeschini R., Mauri A., Livingstone D., Ertl P., Palyulin V.A., Radchenko E.V., Zefirov N.S., Makarenko A.S., Tanchuk V.Y., Prokopenko V.V. (2005). Virtual computational chemistry laboratory--design and description.. J. Comput. Aided Mol. Des..

[8] Tosco P., Balle T. (2011). Open3DQSAR: a new open-source software aimed at high-throughput chemometric analysis of molecular interaction fields.. J. Mol. Model..

[9] Veselinović A.M., Veselinović J.B., Živković J.V., Nikolić G.M. (2015). Application of SMILES notation based optimal descriptors in drug discovery and design.. Curr. Top. Med. Chem..

[10] Toropov A.A., Rallo R., Toropova A.P. (2015). Use of quasi-SMILES and monte carlo optimization to develop quantitative feature Property/Activity relationships (QFPR/QFAR) for nanomaterials.. Curr. Top. Med. Chem..

[11] Gramatica P., Chirico N., Papa E., Cassani S., Kovarich S. (2013). QSARINS: A new software for the development, analysis, and validation of QSAR MLR models.. J. Comput. Chem..

[12] Murrell D.S., Cortes-Ciriano I., van Westen G.J., Stott I.P., Bender A., Malliavin T.E., Glen R.C. (2015). Chemically aware model builder (camb): An R package for property and bioactivity modelling of small molecules.. J. Cheminform..

[13] Wenlock M.C., Carlsson L.A. (2015). How experimental errors influence drug metabolism and pharmacokinetic QSAR/QSPR models.. J. Chem. Inf. Model..

[14] Zhao L., Wang W., Sedykh A., Zhu H. (2017). Experimental errors in QSAR modeling sets: What we can do and what we cannot do.. ACS Omega.

[15] Roy K., Ambure P., Aher R.B. (2017). How important is to detect systematic error in predictions and understand statistical applicability domain of QSAR models?. Chemom. Intell. Lab. Syst..

[16] Steinbeck C., Hoppe C., Kuhn S., Floris M., Guha R., Willighagen E.L. (2006). Recent developments of the chemistry development kit (CDK) - an open-source java library for chemo- and bioinformatics.. Curr. Pharm. Des..

[17] Roy K., Kar S., Das R. (2015). Statistical methods in QSAR/QSPR.. A Primer on QSAR/QSPR Modeling..

[18] Roy K., Kar S., Das R. (2015). QSAR/QSPR Modeling: Introduction.. A Primer on QSAR/QSPR Modeling..

[19] Veerasamy R., Rajak H., Jain A., Sivadasan S., Varghese C.P., Agrawal R.K. (2011). Validation of QSAR Models - Strategies and Importance.. Int. J. Drug Des. Discovery.

[20] Todeschini R., Ballabio D., Grisoni F. (2016). Beware of Unreliable Q(2)! A Comparative Study of Regression Metrics for Predictivity Assessment of QSAR Models.. J. Chem. Inf. Model..

[21] Roy K., Kar S., Ambure P. (2015). On a simple approach for determining applicability domain of QSAR models.. Chemom. Intell. Lab. Syst..

[22] Pearlman R.S., Smith K. (1998). Novel software tools for chemical diversity. In 3D QSAR in drug design..

[23] Ma C., Wang L., Xie X-Q. (2011). GPU accelerated chemical similarity calculation for compound library comparison.. J. Chem. Inf. Model..

[24] Bajusz D., Rácz A., Héberger K. (2015). Why is Tanimoto index an appropriate choice for fingerprint-based similarity calculations?. J. Cheminform..

[25] Wu Y., Li J., Wu J., Morgan P., Xu X., Rancati F., Vallese S., Raveglia L., Hotchandani R., Fuller N., Bard J., Cunningham K., Fish S., Krykbaev R., Tam S., Goldman S.J., Williams C., Mansour T.S., Saiah E., Sypek J., Li W. (2012). Discovery of potent and selective matrix metalloprotease 12 inhibitors for the potential treatment of chronic obstructive pulmonary disease (COPD).. Bioorg. Med. Chem. Lett..

[26] Shamsara J., Shahir-Sadr A. (2014). A predictive HQSAR model for a series of tricycle core containing MMP-12 inhibitors with dibenzofuran ring.. Int. J. Med. Chem..

